# Pilot Testing of Calcium Looping at TRL7 with CO_2_ Capture Efficiencies toward 99%

**DOI:** 10.1021/acs.energyfuels.4c02472

**Published:** 2024-07-24

**Authors:** Borja Arias, Yolanda Alvarez Criado, Alberto Méndez, Paula Marqués, I. Finca, J. Carlos Abanades

**Affiliations:** † CSIC-INCAR, Francisco Pintado Fe, 26, Oviedo 33011, Spain; ‡ HUNOSA, Avenida de Galicia 44, 33055 Oviedo, Spain

## Abstract

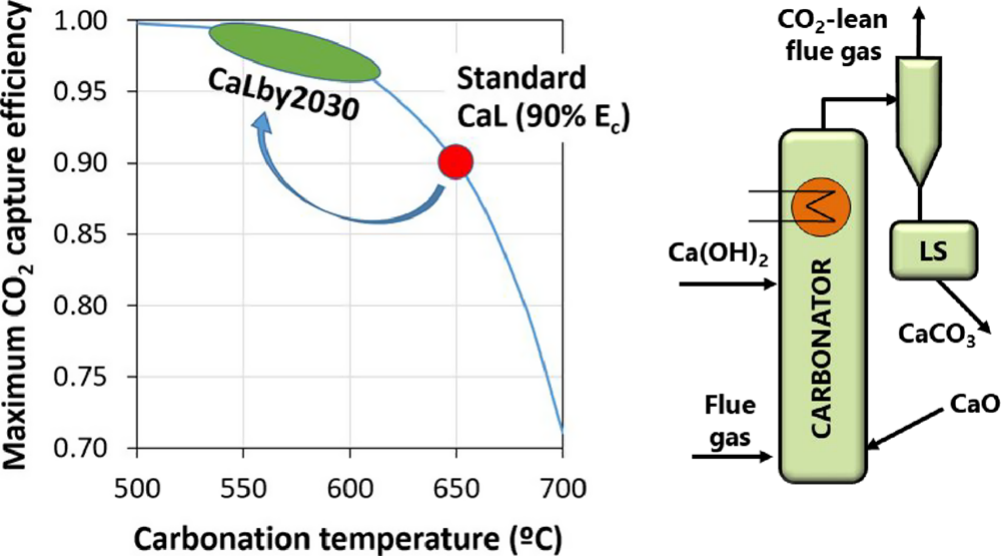

Postcombustion CO_2_ capture by calcium looping using circulating fluidized bed technology, CFB-CaL, is evolving to tackle industrial sectors that are difficult to decarbonize. In addition to the known advantages of CFB-CaL (i.e., retrofittability and competitive energy efficiencies and cost), the fuel flexibility by using renewable biomass in the oxy-fired CFB calciner and the possibility to reach extremely high CO_2_ capture efficiencies in the carbonator are demonstrated in this paper. Results from the latest experimental campaigns in the TRL7 CFB-CaL pilot of the La Pereda are reported, treating over 2000 N m^3^/h of flue gases in the carbonator with a firing capacity of biomass pellets up to 2 MW_th_ in the oxy-fired calciner. A new strategy to reach high CO_2_ capture efficiencies (above 99% in some cases) in the carbonator has been tested. This involves decoupling the carbonator in two temperature zones by cooling the solids-lean top region to below 550 °C and ensuring that a sufficient flow of active CaO reaches such a region.

## Introduction

Calcium looping, CaL, is a CO_2_ capture technology that uses CaO as the CO_2_ sorbent. Shimizu et al.^1^ published the first conceptual process in 1999 for postcombustion CO_2_ capture in coal power plants using bubbling fluidized bed reactors. In this process, a carbonator operated at temperatures around 650 °C is interconnected with a coal-fired calciner operated under oxy-combustion conditions at 950 °C. One advantage of such a process is that the heat required to drive the endothermic calcination of CaCO_3_ can be effectively recovered at boiler temperatures in the carbonator, thus allowing for low energy penalties.^1,2^ Around that time, research on calcium looping and chemical looping combustion was initiated at CSIC. Under an European project,^3^ there was rapid progress in the understanding of key phenomena at the particle level in these high temperature solid looping systems, as reported in some seminal papers by Prof. Adánez′s group on the selection of oxygen carriers for chemical looping combustion.^4^

**Figure 1:**
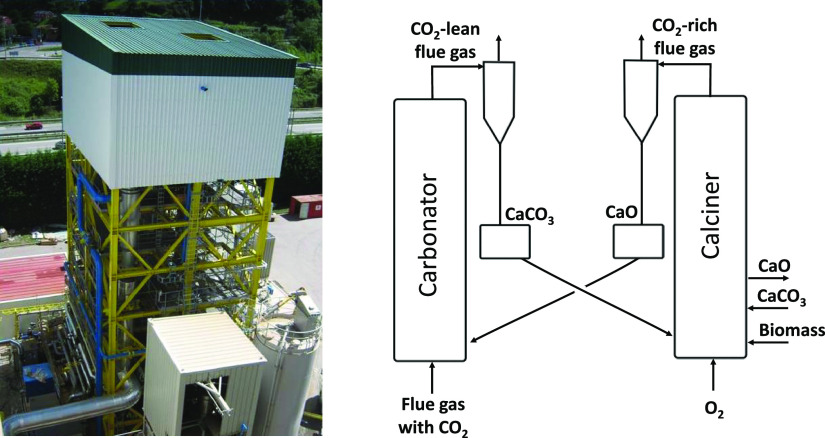
General view of the La Pereda pilot plant (left) and the general scheme of a CFB-CaL system (right).

Many process, reactor, and material alternatives involving CaL reactions have been published in the past few years, with about 50 review papers published on the CaL topic.^5−7^ Tan et al.^8^ have recently compiled the characteristics and status of pilot testing facilities from 1 kW_th_ to 20 MW_th_, with the largest pilot reporting results rated at the MW_th_ scale.^9−17^ From 2009, the pilot plants operated by CSIC have been characterized by the use of two interconnected circulating fluidized bed (CFB) reactors (Figure 1, right),^10,18^ as it was early recognized that postcombustion CaL using this type of reactors was the suitable configuration to deal with the large volumetric flows of gases in and out of the reactors and solids circulation between reactors. CFB reactors benefit from their similarity with circulating fluidized bed combustion (CFBC) boilers in terms of material characteristics, solid circulation rates, gas flow rates, and a combustion atmosphere (facilitating a safe interconnection). Over 6000 h of operating experience has been achieved so far in CFB-CaL pilots run by CSIC.^10,13,17−20^

The use of high solid circulation rates in CFBs can mitigate, if not make it irrelevant, the known decay in CO_2_-carrying capacity of CaO over many cycles of carbonation and calcination.^21,22^ This is because experimental evidence from the pilots has confirmed that, after multiple calcination–carbonation cycles, the CaO resulting from calcination of natural limestone provides sufficient CO_2-_carrying capacity, *X*_ave_, to achieve high capture efficiencies as long as the solid circulation rates between carbonator and calciner are adjusted accordingly (i.e., with a sufficient molar flow of active CaO to at least match the targeted flow of captured CO_2_).^10,15,18,20^ Such features of CFBs facilitated a rapid scale up of CFB-CaL technology between 2010 and 2012, from the results of the first 30 kW_th_ pilot^23^ to the results from the 1.7 MW_th_ of the La Pereda pilot plant used in this work.^10^ For the scaling-up of this CFB-CaL pilot facility, it was critical the support of the pilot plant co-owners HUNOSA and ENDESA, which were interested at the time in decarbonizing coal power plants,^24,25^ as well as the support of Foster Wheeler Energia (what is now Sumitomo SHI FW), which provided their expertise in CFBC power plant technology.

The rapid decline in Europe of coal-based power generation in the past decade represented a decisive setback to the scale-up plans of CFB-CaL technology. However, under a recent Horizon Europe R&D project,^26^ new plans are in place to adapt CFB-CaL technologies to major industrial sectors (i.e., cement and steel) and future power generation combustion systems using residual biomass and waste. As commercial CFBC boilers do, CFB-CaL systems can, in principle, use a wide variety of fuels in the calciner, including biomass or other carbon-neutral fuels. Some works have reported in the literature results from pilots using biomass and solid recovery fuel in the oxy-fired calciner.^27−29^ The use of this kind of fuel implies that CFB-CaL systems can result in negative values if the CO_2_ evolved from the calciner is permanently stored or provide a source of renewable CO_2_ for the manufacture of CO_2_-based synthetic products.^30,31^

**Figure 2:**
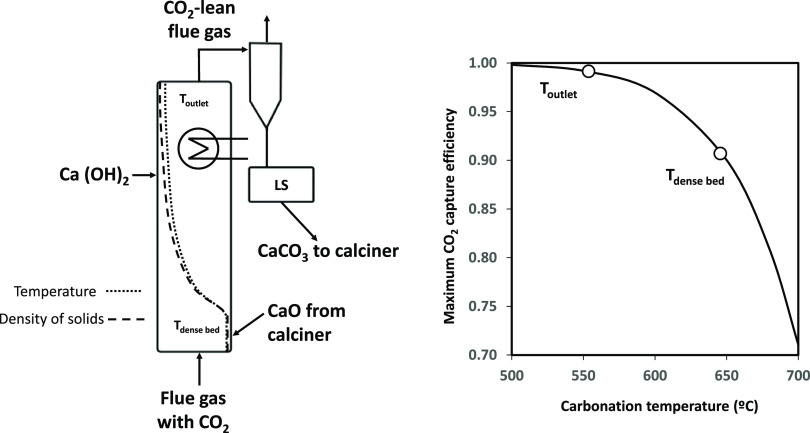
(Left) Basic scheme of the carbonator configuration targeted to achieve high CO_2_ capture efficiencies. (Right) Maximum CO_2_ capture efficiency in the carbonator of a CaL system as a function of temperature (for an inlet gas with 12%_v_ CO_2_, using the equation of Baker for the equilibrium of CO_2_ on CaO^36^).

On the other hand, reaching very high capture efficiencies (i.e., capturing over 99% of CO_2_) in postcombustion systems at atmospheric pressure is challenging due to the reduced driving force of CO_2_ toward the exit of the capture device, whether this is an absorption tower, a bed of solid sorbents, or a membrane. The challenge is not different at the exit of a CFB-CaL carbonator, as most of the active part of the CaO has reacted already with CO_2_ in the dense region at the bottom of the carbonator.^32^ In principle, this makes it difficult for the particles to further react with residual CO_2_ diluted in the flue gases at the top of the carbonator. However, early experiments by Fan and co-workers,^33^ and results from a drop tube carbonator reported elsewhere,^34^ have demonstrated that nascent CaO (in particular if this comes from the in situ decomposition of Ca(OH)_2_) has an extremely high reactivity toward CO_2_ in a wide range of carbonation temperatures. Therefore, a variant of the CFB-CaL system of Figure 1 has been proposed by CSIC,^35^ involving a carbonator with enhanced cooling capabilities to promote enhanced carbonation at the top of the reactor as represented in Figure 2-left (for simplicity, other heat transfer equipment in the CaL system is omitted).

The purpose of the additional heat exchange represented in Figure 2-left is to reduce the temperatures in that region to below 600 °C, so that the CO_2_–CaO equilibrium allows for deeper decarbonization of the flue gases. For example, as represented in Figure 2-right, for a typical flue gas inlet of 12%_v_ CO_2_, thermodynamics allows 99% capture efficiency when the carbonator is designed to operate at 550 °C. However, this comes with some trade-offs when compared to state-of-the-art CFB-CaL systems, usually designed to achieve a CO_2_ capture efficiency of 90% with the carbonator operating at 650 °C. First, the heat demand in the oxy-fired calciner would increase, as more energy would be needed to heat up the solids coming from the carbonator. Another drawback is the decrease in the maximum CO_2-_carrying capacity of CaO as the carbonation temperature reduces^37^ which will demand higher solid circulation rates to transport the CO_2_ from the carbonator to the calciner. Finally, the lower temperatures in the carbonator will slightly reduce the energy efficiency of the steam cycle recovering energy from the carbonator and therefore increase the required area of the boiler tubes. To counteract these impacts, the carbonator of Figure 2-left requires the generation of an axial temperature gradient to produce a temperature drop of about 100 °C in the top region. This can be achieved through the combined effect of a second heat exchanger, by means of the injection of the makeup flow of limestone at that point or even by quenching with liquid water. With this approach, high carbonation temperatures will be maintained in the dense bottom region of the CFB carbonator (similar to a standard carbonator configuration), where the intense solid mixing and good gas–solid contact will allow most of the carbonation and heat extraction to take place. In any case, to approach equilibrium in the carbonator exit region, it is important that the CaO material entrained upward to that cooled region has sufficient reactivity toward CO_2_.

As part of the recent Horizon Europe R&D project,^26^ many small retrofits have been implemented on the La Pereda CFB-CaL pilot plant to make it operational again and to test for the first time in this pilot the oxy-combustion of wood pellets in the calciner as well as the new process variant in the carbonator proposed above. The purpose of this paper is to report a new set of results of a three week-long experimental campaign that demonstrates at TRL7 the viability of enhanced carbonation by adjusting the temperature profile, as shown in Figure 2, with 99% CO_2_ capture efficiencies in the carbonator while operating the calciner under oxy-combustion of biomass.

## Experimental Section

The results presented in this work have been obtained during several experimental campaigns carried out in the La Pereda 1.7 MW_th_ pilot plant shown in Figure 1-left (a more detailed description of this facility can be found elsewhere^10,19^). This pilot can treat a slip of the flue gas produced in an existing CFB power plant. However, due to a long shutdown of this power plant, flue gas was not available, and the gas fed into the carbonator during these experimental campaigns was produced by mixing air with CO_2_ coming from a cryogenic tank. The pilot includes two circulating fluidized bed reactors, a carbonator, and a calciner, with a height of 15 m and internal diameters of 0.65 and 0.75 m, respectively, that are interconnected through two loop seals. Gas velocities inside the carbonator and calciner are around of 2–6 m/s, typical of CFB boilers. The temperature in the carbonator can be adjusted using four water-cooled bayonet tubes installed in the upper part of the reactor, whose insertion length can be modified to change the cooling surface area. The calciner has an inlet at the bottom for the feeding of fuel and limestone. The fuel can be burned under oxy-firing conditions by feeding a mixture of O_2_/CO_2_ coming from cryogenic gas tanks. The composition of the gaseous streams entering and leaving the reactors is measured continuously using four gas analyzers. The composition of the solids circulating in the system is determined periodically by taking solids samples at different ports located along the facility.

Several small modifications were carried out before the experimental campaigns in order to make the pilot plant operational again after a long shutdown and to adapt it to the new operation conditions. These were mainly related to the upgrade of the gas analysis system and some repairments in the solid circulation system. During these experimental campaigns, pellets of biomass were used as fuel (50.7%C_wt_, 6.0%H_wt_, 34.6%O_wt_, 8.4%H_2_O_wt_, LHV = 19.1 MJ/kg) as the existing fuel feeding system can handle this kind of fuels after tuning the operational parameters. Table 1 summarizes the main operation conditions during the tests reported in this work.

**Table 1:** Range of Operating Conditions and Main Variables During the Tests Reported in This Work

average temperature in the carbonator, *T*_CB_	°C	530–660
superficial velocity at the carbonator	m/s	2.5–4.0
inventory of solids in the carbonator	kg/m^2^	350–650
inlet CO_2_ volume fraction at the carbonator inlet		0.03–0.14
maximum CO_2_ carrying capacity of the solids, *X*_ave_		0.10–0.40
average temperature in the calciner, *T*_CC_	°C	840–945
superficial velocity at the calciner	m/s	3.0–5.5
inventory of solids in the calciner	kg/m^2^	50–250
O_2_ volume fraction in the oxidant		0.30–0.40
biomass mass flow rate	kg/h	250–450
CO_2_ capture efficiency, *E*_CO2_		0.60–0.995

## Results and Discussion

**Figure 3:**
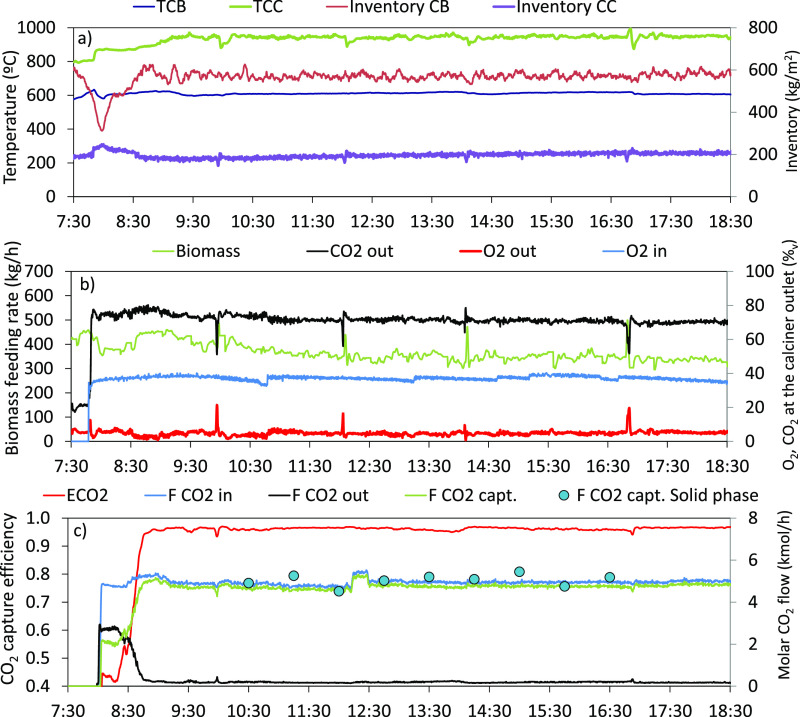
Example of an experimental CO_2_ capture test with oxy-fuel combustion of biomass in the calciner.

Initial tests were aimed at testing the pilot plant operability by using biomass pellets as fuel. As an example, Figure 3 shows the main variables during a typical experiment, in which the calciner was operated under oxy-fuel conditions. After an initial period of preheating the pilot and achieving temperatures above 800 °C in the calciner (not shown in the graph for simplicity), the combustion conditions in the reactor are switched from air to oxy-fuel with an average oxygen concentration of 37%v in the oxidant (at 7:40). As a result, there is a sharp increase in the CO_2_ concentration at the outlet of the calciner (see Figure 3b). Regarding the carbonator, the gas fed into the reactor is changed from air to a flue gas with a CO_2_ concentration of 11.0%v at 8:00 (Figure 3c). After a short period, the average temperature in the calciner and the carbonator is stabilized and maintained at values around 935 and 615 °C, respectively. This is achieved by burning an average biomass flow rate of 365 kg/h (resulting in a thermal input of 1.94 MW_th_) with an excess of oxygen at the outlet of the calciner below 5%v. From this point, the gas velocities in the reactors are maintained constant at 3.3 and 3.8 m/s in the carbonator and calciner, respectively. Under these conditions, the circulation of solids between reactors is 1.4 kg/s, with solid inventories of 570 and 195 kg/m^2^ in the carbonator and calciner, measured from the pressure drop readings.

Figure 3c shows the flow of CO_2_ captured in the carbonator, which can be estimated using two methods in this facility: from the mass balance on the gas phase (measurements of total gas flows and CO_2_ contents in and out of the carbonator, solid green line in Figure 3c) and from the mass balance in the circulating solids. The latter is estimated from the product of the molar flow of CaO entering the reactor (*F*_Ca_) and the increment in the carbonate content measured on solid samples taken from the inlet and outlet of the reactor (*X*_carb_ – *X*_calc_) (blue dots in Figure 3c). As can be seen, there is good agreement between both methods, yielding a flow of captured CO_2_ of about 4.6 kmol/h. This results in an average CO_2_ capture efficiency of 0.96 during this period.

**Figure 4:**
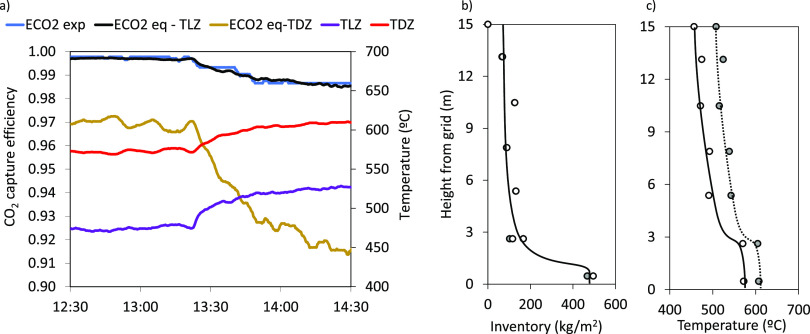
(a) Example of the effect of the carbonator temperature on the carbonation efficiency; (b,c) inventory of solids and temperature profile along the carbonator (gray dots: from 12:30 to 13:20; white dots: from 14:00 to 14:30).

After these initial tests, several experiments were carried out to demonstrate the viability of the approach shown in Figure 2 to achieve a CO_2_ capture efficiency above 0.99. For this purpose, the carbonator was operated with a wide range of temperatures, as shown in Table 1. Moreover, most of these tests were carried out with a high makeup flow of CaCO_3_ fed into the calciner to operate the system with a sorbent with high CO_2_ carrying capacities (*X*_ave_) ensuring a sufficient flow of active CaO material in the upper zone of the carbonator. Figure 4 shows an example of an experimental period aimed at achieving extremely high CO_2_ capture efficiencies. For this specific test, the low temperatures in the carbonator were obtained by feeding a flue gas with a low CO_2_ concentration (5.8%v) so as to reduce the heat generated by the carbonation and also by maximizing the heat extraction with the four water-cooled bayonet tube heat exchangers fully inserted into the carbonator. In addition, the conditions were adjusted to operate with a high excess of active sorbent (*F*_Ca_*X*_ave_) with respect to the molar CO_2_ flow (*F*_CO2_) fed into the carbonator (*F*_Ca_*X*_ave_/*F*_CO2_ of 1.8). The inventory of solids in the carbonator during this test was 495 kg/m^2^, which presented a typical distribution in CFB reactors, with the presence of a dense zone in the bottom of the reactor and a lean zone in the upper part (see Figure 4b). As shown in Figure 4a, from 12:30 to 13:20, the temperature in the carbonator was maintained constant. The temperature profile along the reactor during this period is shown in Figure 4c (white dots). As can be seen, there is a homogeneous temperature of around 570 °C in the bottom dense zone. From this point, there is a sharp drop in the temperature profile with an average value of 475 °C in the upper lean zone. During this initial period, a CO_2_ capture efficiency of 0.995 was achieved in the carbonator, as shown in Figure 4a. Then, the bayonet tubes in the carbonator were partially removed to reduce heat extraction at 13:20. As a result, the temperature in the reactor increased, especially in the upper lean zone (from 475 up to 525 °C), resulting in a decrease in the CO_2_ capture efficiency, as can be seen in Figure 4a.

Using the temperature profiles along the carbonator, two different average zones with their respective average temperatures were defined to facilitate the data interpretation that follows. One corresponds to the average temperature in the bottom dense zone of the carbonator, *T*_DZ_ (from 0 to 3 m above the grid), and the other to the temperature in the upper lean zone, *T*_LZ_ (from 8 to 15 m above the grid). These two temperatures, represented in Figure 4a, were used to calculate the maximum capture efficiency that can be achieved considering the CO_2_ partial pressure given by the equilibrium (*E*_CO2 eq-TLZ_ and *E*_CO2, eq-TDZ_, respectively in Figure 4a). As can be seen, the experimental capture efficiency matches the maximum efficiency given by the equilibrium considering the temperature in the upper lean zone, while it clearly exceeds that limited by the temperature in the dense bed. This can be seen clearly during the second period operating at higher temperatures. In this case, the experimental CO_2_ capture efficiency was 0.985, while the temperature in the dense bed only allows for a maximum capture of 0.915.

To interpret the observations presented above in a more quantitative manner, a basic 0D reactor model has been used, adapted from previous works where it was developed for more standard CFB-CaL experiments.^10,20,38^ The use of more sophisticated comprehensive 3D models^32^ to analyze in detail the impact of the heat balances and the mixing of solids in the carbonator on the carbonator performance has been considered outside the scope of this work but will be addressed later in the CaLby2030 project when more data are available.^26^

**Figure 5:**
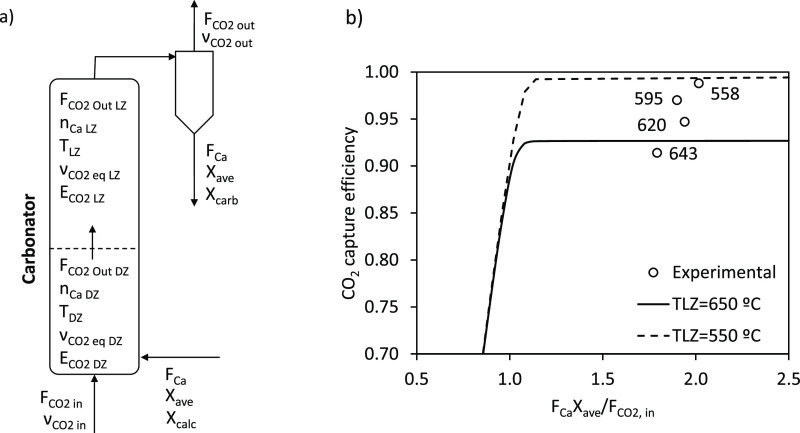
(a) Scheme of the CFB carbonator with the main variables involved. (b) Effect of the active sorbent/CO_2_ ratio on the CO_2_ capture efficiency for two different temperatures in the lean zone (lines) and the experimentally obtained i (dots).

The basic 0D model assumes that the solid phase behaves as a perfect mix reactor, while the gas phase can be considered as a plug flow reactor. It also assumes that the reaction rate of the particles is constant until the maximum carbonate conversion (*X*_ave_) is achieved, which becomes zero after that point.^20,39^ The CO_2_ capture (*E*_carb_) in the carbonator is defined as
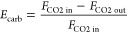
1where *F*_CO2 in_ and *F*_CO2 out_ are the molar flow entering and leaving the carbonator, respectively. According to the temperature and solid profiles, as shown in Figure 4, the carbonator is assumed to be composed of two zones, one dense zone located at the bottom part and one lean zone in the upper part, operating at two different temperatures, *T*_DZ_ and *T*_LZ_. It can be argued that the mixing of solids between these two zones is sufficiently intense to assume that the whole inventory of solids in the carbonator is perfectly mixed with respect to the solid composition, but sufficiently limited to allow the observed difference in temperatures between the two zones when the cooling capacity in the upper part is sufficiently intense. On the other hand, the gas phase is considered as two plug flow reactors connected in series. With these simplifying assumptions, the following CO_2_ mass balance closure can be solved for each zone (see also Figure 5a):
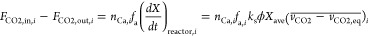
2where *F*_CO2in,*i*_ is the molar flow of CO_2_ entering each zone, *F*_CO2out,*i*_ is the molar flow of CO_2_ leaving each zone, *n*_Ca,*i*_ is the inventory of solids, *k*_s_ϕ is an apparent constant reaction rate which has a value of 0.36 s^–1^ for the limestone used in this work,^10,18^ is the average CO_2_ concentration, and *f*_a,*i*_ is the fraction of active solids which is calculated as follows, from the residence time distribution curve of a well-mixed reactor:
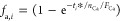
3where *n*_Ca_ is the total inventory of solids, *F*_Ca_ is the molar flow rate of calcium entering the carbonator, and t_i_^*^ is the time needed to achieve the maximum CO_2_ carrying capacity under the reaction conditions in each zone (*t*_i_^*^ = (*X*_ave_ – *X*_calc_)/(*k*_s_ϕ(ν_CO2_– ν_CO2 eq_))).

Figure 5b presents the results obtained during an experiment in which the carbonator was modified, while an average value of the ratio *F*_Ca_*X*_ave_/*F*_CO2,in_ of 1.9 was maintained. The inlet CO_2_ concentration into the carbonator during this period was 14.2 vol % with a molar flow of 5.7 kmol/h. The total inventory of calcium solids in the carbonator was 675 kg/m^2^. The dots in this figure present the CO_2_ capture achieved for different temperatures in the lean zone (*T*_LZ_ values in °C are shown in the figure as labels). As can be seen, reducing the temperature by 85 °C in the lean zone increases the CO_2_ capture efficiency from 0.91 up to 0.99. This graph also includes two lines that have been calculated using the methodology described above for two cases that consider the same temperature in the dense bed (*T*_DZ_ = 650 °C) and two temperatures in the lean zone (*T*_LZ_ = 550 and 650 °C). For this calculation, it has been assumed that 70% of the total inventory is found in the dense zone of the carbonator and an average molecular weight of the solids of 60 g/mol, which is in agreement with the experimental measurements. Then, eq 2 is solved simultaneously for both zones, so the molar flow of CO_2_ and the CO_2_ concentration at the outlet of the dense zone is equal to the molar flow of CO_2_ and the CO_2_ concentration at the inlet of the lean zone, for different ratios of *F*_Ca_*X*_ave_/*F*_CO2,in_.

As can be seen, the experimental values fall reasonably inside the region between the two cases. The results shown in this graph indicate that the improvement in the CO_2_ capture efficiency is only noticeable when there is an excess of active sorbent flow entering the carbonator (i.e., *F*_Ca_*X*_ave_/*F*_CO2_ > 1.5).

**Figure 6:**
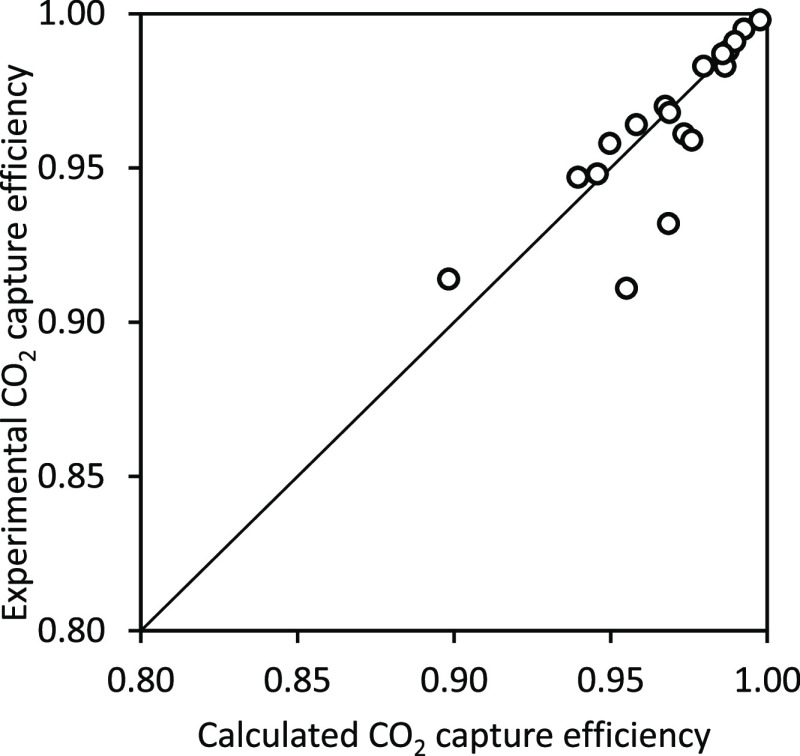
Comparison of the experimental CO_2_ capture efficiency and the calculated values.

Finally, Figure 6 compares the experimental CO_2_ capture efficiency and the values calculated with the model including a wider range of operation conditions (e.g., inventory of solids, temperature, CO_2_ inlet concentrations, etc.). The data shown in this figure correspond to experimental periods in which there was sufficient flow of active sorbent entering the carbonator (*F*_Ca_*X*_ave_/*F*_CO2_ > 1.3). A reasonable agreement can be observed between both values.

These results highlight the need to fulfill two simultaneous conditions to achieve deep decarbonization in the carbonator of a CFB-CaL system: a temperature around 550 °C (or below) in the upper part of the reactor to overcome thermodynamic limitations and a sufficient flow rate of active sorbent in the upper region of the carbonator. As indicated above, this second requirement has been achieved in this work by using large makeup flows of limestone in order to ensure the presence at the top of the carbonator of an excess of active CaO a sorbent with a high CO_2_ carrying capacity (*X*_ave_). For most postcombustion applications (except in cement or lime plants where the CaO-rich purge is a coproduct and high makeup flows will be used), this approach could be too demanding in terms of high operating costs. However, as discussed in Figure 2, we have planned retrofits in the La Pereda pilot plant that will include the injection of a Ca(OH)_2_ polishing flow, allowing it to reach such high CO_2_ capture rates with moderate values of makeup flow of limestone.

## Conclusions

A novel strategy to increase the CO_2_ capture efficiency in the carbonator of a calcium looping system using CFB reactors has been experimentally tested in this work. This consists of the cooling of the upper part of the carbonator to create a low temperature zone and avoid the equilibrium limits on the minimum achievable CO_2_ concentration. For this purpose, experiments in a TRL7 CFB-CaL pilot have been carried out operating the calciner under typical oxy-fuel conditions burning biomass at a rate of 2.0 MWth and high makeup flows of limestone (*F*_Ca_*X*_ave_/*F*_CO2_ > 1.3). The results confirm that it is possible to reach CO_2_ capture efficiencies above 0.99 by ensuring that the temperature at the outlet of the carbonator is sufficiently low (<550 °C) and there is enough sorbent available in the cooled zone. The results have been successfully interpreted using a basic model that considers two reaction zones in the carbonator. Future retrofits in the La Pereda pilot plant are planned to feed Ca(OH)_2_ in the upper part of the carbonator, to reach CO_2_ capture rates over 99% without the need for large makeup flows of limestone.
